# Community Engagement and Data Disclosure in Environmental Health Research

**DOI:** 10.1289/ehp.1510411

**Published:** 2016-02-01

**Authors:** Erin N. Haynes, Sarah Elam, Roxanne Burns, Alonzo Spencer, Elissa Yancey, Pierce Kuhnell, Jody Alden, Mike Walton, Virgil Reynolds, Nicholas Newman, Robert O. Wright, Patrick J. Parsons, Meredith L. Praamsma, Christopher D. Palmer, Kim N. Dietrich

**Affiliations:** 1 Department of Environmental Health, College of Medicine, University of Cincinnati, Cincinnati, Ohio, USA; 2 Center for Clinical and Translational Science and Training, University of Cincinnati, Cincinnati, Ohio, USA; 3 Kent State University at East Liverpool, East Liverpool, Ohio, USA; 4 Save Our County, Inc., East Liverpool, Ohio, USA; 5 Department of Journalism, College of Arts and Sciences, University of Cincinnati, Cincinnati, Ohio, USA; 6 Division of Epidemiology and Biostatistics, Cincinnati Children’s Hospital Medical Center, Cincinnati, Ohio, USA; 7 Division of General and Community Pediatrics, Cincinnati Children’s Hospital Medical Center, Cincinnati, Ohio, USA; 8 Department of Preventive Medicine, Division of Environmental Health, Mount Sinai Hospital, School of Medicine, New York, New York, USA; 9 Laboratory of Inorganic and Nuclear Chemistry, Wadsworth Center, New York State Department of Health, Albany, New York, USA; 10 Department of Environmental Health Sciences, School of Public Health, University at Albany, Albany, New York, USA; * Deceased.

## Abstract

Summary: Federal funding agencies increasingly support stakeholder participation in environmental health studies, and yet there is very little published research on engagement of community members in the development of data disclosure (DD) strategies. The Ohio Environmental Protection Agency reported airborne manganese (Mn) concentrations in East Liverpool, Ohio, 30 times higher than the reference concentration, which led to an academic–community research partnership to address community concern about Mn exposure, particularly among children. Children and their families were recruited to participate in a pilot study. Samples of blood and hair were collected from the children and analyzed for metals. DD mechanisms were developed using an iterative approach between community and academic partners. Individual DD letters were mailed to each participating family, and a community meeting was held. A post-meeting survey was administered to gauge community perception of the DD strategies. The purpose of this article is to demonstrate the effectiveness of engaging community partners in the conduct of environmental health research and in the development of DD strategies for individuals and the community at large. Scientists should include community partners in the development of DD strategies to enhance translation of the research findings and support the right of study participants to know their individual results.

## Introduction

Federal funding agencies increasingly support stakeholder participation in environmental health research (NRC 2012; O’Fallon and Dearry 2002), as noted by the 2014 Institute of Medicine Roundtable (http://iom.nationalacademies.org/Activities/Environment/EnvironmentalHealthRT/2014-MAR-19.aspx) on sharing environmental health research data. The National Institute of Environmental Health Sciences (NIEHS) supports raising awareness levels of environmental health literacy through community–academic partnerships and community-engaged research (Finn and O’Fallon 2015). Community-based participatory research (CBPR) is a research methodology that encourages engagement of the study population throughout the research process, from inception to receiving the results (Israel et al. 2001; Minkler and Wallerstein 2008). Incorporating CBPR principles in environmental health science research has been practiced for more than a decade (Arcury et al. 2001; Wing et al. 2008; Brown et al. 2012; Haynes et al. 2011), and yet there is little to no published research on the engagement of community stakeholders in the development of data disclosure (DD) strategies (i.e., providing research results at the individual level and community level).

East Liverpool, Ohio, is a rural, underserved Appalachian community. It is situated along the Ohio River with a population of 10,951; of these, 91% are Caucasian (non-Hispanic white), and 30% of persons are below the federal poverty level as compared with 16% of persons residing in the state of Ohio (U.S. Census Bureau 2015). The city of East Liverpool has experienced a dramatic decline in population since 1970 when there were 20,020 residents (U.S. Census Bureau 2015). During the 2010 academic year, the East Liverpool School District reported a higher percentage of students in special education (19%) than did the state of Ohio (13%) (Ohio Department of Education 2011). In addition to economic difficulty, the community is faced with potentially significant environmental exposures. The Ohio Environmental Protection Agency (Ohio EPA) identified airborne manganese (Mn) concentrations from an air sampling station in East Liverpool that was 30 times higher than the U.S. Environmental Protection Agency (EPA) reference concentration of 0.05 μg/m^3^ (Ohio EPA 2010; U.S. EPA 1993). Based on the airborne Mn concentrations at the three air monitoring sites in East Liverpool, the Ohio EPA reported a non-cancer hazard index (HI) ranging from 4 to 32; an HI above 1.0 is considered “potentially unacceptable and merits further investigation” (Ohio EPA 2010). The Ohio EPA identified the S.H. Bell Company, a ferromanganese processing facility located 200 ft from residential areas, as the primary contributing source of the high Mn levels in the air found in East Liverpool (Ohio EPA 2010). The company handles and distributes metals, minerals, and semi-finished industrial materials.

Mn is an essential trace element needed for normal growth and development, particularly for the brain; however, in excess it can be neurotoxic. Inhaled Mn is capable of crossing the blood–brain barrier and accumulating in the brain (Dorman et al. 2002); it is known to lead to adverse neurological outcomes in highly exposed adults and children (Lucchini et al. 2012; Rugless et al. 2014; Haynes et al. 2015). Thus, an academic–community partnership was formed between scientists and the residents of East Liverpool to address community concerns about potential exposure to airborne Mn.

In this article, we illustrate how community stakeholders within a rural Appalachian community were engaged in the design and conduct of an environmental health research study and in the development of DD strategies at the individual level and at the community level.

## Materials and Methods

### Development and Conduct of the Pilot Study

In response to the Ohio EPA 2010 Air Toxics Report (Ohio EPA 2010), the superintendent of the East Liverpool Public Schools publically requested, during a local Board of Health meeting, that “hair metal level tests” and “follow-up neuropsychological tests” be conducted on school-age children (McElwain 2010). After being contacted by the superintendent regarding the possibility of conducting these tests, a member of the research team (E.N.H.) began to assemble a group of community members to participate in the study and to help guide the conduct of the study. Community members were identified following conversations with the superintendent. Representatives from the community included four coauthors (A.S., M.W., V.R., and R.B.) of this article, faculty and students from Kent State University at East Liverpool and the local school districts, and concerned community residents, including parents of young children. Although several meetings were conducted in East Liverpool, most communication among the research team was through conference calls. The academic members of the team, all of whom are coauthors on this article, included individuals with expertise in metals epidemiology, biomarker analyses, data management, community outreach and engagement, pediatric medicine, and journalism. Community representatives and other community members provided expertise and insight into the community and the target population as the team developed the pilot study and the DD strategies both at the individual level and community level.

Children who were 4–17 years of age and who resided in East Liverpool, Ohio, or the surrounding area (e.g., Wellsville, OH; Newell and Chester, PA) were recruited to participate in the East Liverpool Pilot Study. Siblings from the same household were eligible to participate. Recruitment postcards were sent home with children through schools, and advertisements were aired on local radio stations and printed in local newspapers (Baligush 2011; McKinnon 2011). The pilot study was conducted 4–5 November 2011 on the campus of Kent State University at East Liverpool. The University of Cincinnati institutional review board reviewed and approved the study. All parents provided written informed consent, and each child provided written assent. Parents were asked questions about the participating child’s residential and health history and socioeconomic demographics. The child’s height and weight were also measured. All data were collected on the same day.

### Specimen Collection and Analyses

Hair samples were analyzed at the Channing Trace Metals Laboratory, Brigham and Women’s Hospital, Harvard T.H. Chan School of Public Health, Boston, Massachusetts, using methods previously described (Wright et al. 2006). In brief, samples were collected and analyzed according to the guidelines of a similar study in Marietta, Ohio (Haynes et al. 2015).

### Development of the Data Disclosure Strategies

A total of 106 children, representing 64 households, participated in the study (Table 1). Child participants were 4–17 years of age, and the average age was 10 years old. The geometric mean for blood Mn was 9.9 µg/L (SD ± 1.3 µg/L) based on samples from 76 children, and the geometric mean for hair Mn was 715 ng/g (SD ± 2.6 ng/g) based on samples from 93 children.

**Table:** Characteristics of East Liverpool, Ohio, study participants (*n* = 106)

Characteristic	Mean ± SD (range) or *n* (%)
Child Measures
Age (years)	10.2 ± 3.1 (4–17)
Child’s sex
Female	46 (43)
Male	60 (57)
Race/ethnicity
Caucasian (non-Hispanic white)	97 (92)
African American	5 (5)
Hispanic	3 (3)
Native American	1 (1)
BMI	21.6 ± 6.7 (12.7–43)
Medical History
Asthma	39 (37)
Allergies	38 (36)
ADD or ADHD	27 (25)
Autism spectrum disorder	3 (3)
Recommended for special education	24 (23)
Biomarkers^*a*^
Hair Mn (ng/g), *n* = 93	715 ± 2.6 (101–24,923)
Blood Mn (µg/L), *n* = 76	9.9 ± 1.3 (5.9–18.4)
Blood Pb (µg/dL), *n* = 76	1.0 ± 1.7 (0.3–3.2)
Blood Hg (µg/L), *n* = 76	0.1 ± 2.1 (0.02–0.8)
Blood Cd (µg/L), *n* = 76	0.1 ± 1.7 (0.05–1.0)
Household Measures
Household income, *n* = 64	
< $20,000	34 (53)
$20,000–$40,000	17 (27)
> $40,000	13 (20)
Parent Education, *n* = 62
8th grade	2 (3)
High school	23 (37)
Some college	16 (26)
Associate’s Degree	19 (31)
Bachelor’s Degree	2 (3)
Abbreviations: ADD/ADHD, attention deficit disorder/attention deficit hyperactivity disorder; BMI, body mass index; Cd, cadmium; Hg, mercury; Pb, lead. ^***a***^Values for biomarkers are geometric means. Method detection limits (MDL): hair Mn = 2 ng/g (all samples ≥ MDL); blood Mn = 1.5 µg/L (all samples ≥ MDL); blood Pb = 0.04 µg/dL (74 of 76 samples ≥ MDL); blood Cd = 0.2 µg/L (4 of 76 samples ≥ MDL); blood Hg = 0.2 µg/L (15 of 76 samples ≥ MDL).	

Biomarker data were discussed in joint meetings with the academic–community scientific team, and the DD strategies were developed with the participation of all team members. Community team members recommended that the DD include easy-to-read graphics that would be simple to interpret, particularly when preparing each participant’s individual DD. To this end, a blood tube design was created with accompanying summative text. The blood tube image underwent an iterative revision process within the academic–community team until consensus was reached. A few representative comments from community team members during this process included the need for an appropriate reading level and the importance of providing a comparison population to help interpret the results (i.e., “How do our results compare to other studies or to other kids in similar environmental circumstances?”). To address this, we compared group-level geometric mean blood Mn (9.9 µg/L) with a cohort of children of similar ages living near a Mn processing plant in Molango, Mexico (geometric mean, 9.7 µg/L) (Hernandez-Bonilla et al. 2011). Once the blood tube design was finalized, a hair Mn image was also created, reviewed, and revised by the academic–community team members. We compared the arithmetic mean for hair Mn concentration from the pilot study with the reported arithmetic mean within a cohort of children of similar ages in Tar Creek, Oklahoma, using the same analytic methods described by Wright et al. (2006). The arithmetic mean for hair Mn level within our cohort (1,419 ng/g) was 3 times higher than the hair Mn levels found in children living near the hazardous waste site in Oklahoma (471 ng/g) (Wright et al. 2006). These images were accompanied by text and incorporated into the pilot research study fact sheet (http://eh.uc.edu/cares/study/research/EL_Fact_Sheet.pdf) and the individual DD.

The individual DD was tailored for each participant (http://eh.uc.edu/cares/study/research/East%20Liverpool%20Participant%20Letter_Updated_EXAMPLE.pdf); it consisted of a cover letter, individual research results, a fact sheet showing the findings of the pilot study, and one-page summaries of each metal prepared by the Agency for Toxic Substances and Disease Registry (ATSDR 2015). The cover letter thanked the family for their participation, provided an overall summary of the study, an invitation to the community meeting, and contact information for the study’s principal investigator and the Cincinnati Children’s Hospital Medical Center’s Pediatric Environmental Health Specialty Unit if they wanted additional information. A blood tube image indicated their blood metal level, the average blood level found in the pilot study sample population, and a national level, if available, for children of similar ages. Each blood tube image was accompanied by a summative text including a brief description of the relationship of their level to the levels observed in U.S. children, potential sources of exposure, and health risks associated with their particular level, if any. The individual DD was reviewed and approved by the University of Cincinnati institutional review board before dissemination.

The community team members indicated that the best mechanism to notify the community of the study’s findings was through a presentation at the local Board of Health and during a community meeting advertised through local news media. A press release was distributed to community-identified media outlets announcing both meetings. The community partners also determined the ideal location for the community meeting was the Slak Shak in Purinton Hall on the campus of Kent State University at East Liverpool.

### Evaluation of Data Disclosure Strategies

Nearly 50 community members, including representatives from local television and print news media, attended the community meeting. The presenter (E.N.H.) described the study, its findings, and possible future directions. Thirty attendees completed a questionnaire designed to evaluate the effectiveness of the DD information (http://eh.uc.edu/cares/study/research/Results%20Communication%20Questionnaire%20Final.pdf). There was an overwhelming positive response as each respondent (100%) indicated that the information presented at the community meeting was clear and understandable. All respondents (100%) also indicated that the fact sheet was easy to read, was helpful to their understanding of the study results, clearly explained the blood metal and hair Mn results, and that the accompanying images made the results more clear.

The respondents were asked to circle the image (blood tube or data plot) that gave them the information that was most easily understood. The majority of respondents (82%) indicated that the blood tube image was more easily understood than the data plot, 11% indicated that they preferred the data plot, and 7% indicated that they preferred both the blood tube image and the data plot. Representative comments about the blood tube image indicated that this image was “much more understandable,” “good for lay people,” “easier to understand because of the colors and visual effect,” and “sticks out more and is easier to read.” Other comments included, “I liked the blood level picture. It made the results very easy to read,” and “the blood tube is direct.” Representative comments about the data plot indicated that it was “too technical for a layman to interpret,” “harder to read,” “difficult to read and understand,” “harder to see results/understand results,” and “better for academic or medical study reports.” Although the majority of the comments reflected a dislike for the data plot, one respondent noted that it “shows a better comparison.”

When asked other ways in which they would like to receive study results, 62% reported newspaper and 12% suggested radio broadcasts. Nine respondents indicated other ways they would like to receive a summary of study results, including Internet (*n* = 4), social media (*n* = 2), direct mailing (*n* = 2), and television (*n* = 1).

## Discussion

To our knowledge, this is one of the first studies to describe the process of engaging community members as part of the scientific team in developing DD communication strategies for individual- and community-level research results. Although data plots, histograms, or graphs are typically used by scientists to communicate among scientists and convey research results to lay audiences, our data demonstrate that outside-the-scientific-box images, such as a blood tube, is more clearly understood than a traditional scientific format, such as a data plot. By incorporating community partners in the development of the DD strategy, the team was able to develop a communication strategy at the appropriate level of environmental health literacy.

Engagement between scientists and community partners is essential to effectively communicate research results (Chavis et al. 1983; Brody et al. 2007). Community members may report their understanding of graphs, charts, and plots when accompanied by legends and a graph-reading guide, yet during informal conversations and formal interviews, it took time for community members to interpret the meanings of graphs, and they sometimes reported a lack of confidence in their ability to interpret the graphs (Brody 2014; Dunagan et al. 2013).

It is well-recognized that researchers have a responsibility to report individual results in a meaningful way (Mikesell et al. 2013; Quandt et al. 2004), and that participants are eager to receive their results (Adams et al. 2011) and an interpretation of the results in terms of what the exposures mean for their health (Morello-Frosch et al. 2009). However, few environmental health research studies have evaluated individual DD materials to assess community understanding of the information. Scientific graphs or tables may not be appropriate for lay audiences, as they require a higher level of scientific literacy to interpret and use them. Considering the importance of health numeracy in the interpretation and use of health information (Ancker and Kaufman 2007; Schapira et al. 2009), it is increasingly important that scientists consider the visual design of their data presentations when communicating health-related numbers to the public (Finn and O’Fallon 2015).

## Conclusion

An academic–community scientific team effectively developed and implemented an environmental health pilot study and DD strategies at the individual and community level. Incorporating community partners at the onset of developing the study and DD strategies resulted in the creation of an outside-the-scientific-box image (e.g., blood tube) to effectively translate biological data to the target audience. Scientists should include community partners from the target population in the development of research and DD strategies in order to enhance the quality of research, to support the rights of the study participants to know their individual results, and to increase environmental health literacy.
